# Improving early detection of Alzheimer’s disease through MRI slice selection and deep learning techniques

**DOI:** 10.1038/s41598-025-14476-0

**Published:** 2025-08-10

**Authors:** Begüm Şener, Koray Açıcı, Emre Sümer

**Affiliations:** 1 Department of Computer Engineering, Başkent University, Ankara, Turkey; 2 Department of Artificial Intelligence and Data Engineering, Ankara University, Ankara, Turkey

**Keywords:** Alzheimer’s disease, Slice selection, Deep learning, Vision transformers, Early Mild Cognitive Impairment, Cognitive neuroscience, Bioinformatics

## Abstract

Alzheimer’s disease is a progressive neurodegenerative disorder marked by cognitive decline, memory loss, and behavioral changes. Early diagnosis, particularly identifying Early Mild Cognitive Impairment (EMCI), is vital for managing the disease and improving patient outcomes. Detecting EMCI is challenging due to the subtle structural changes in the brain, making precise slice selection from MRI scans essential for accurate diagnosis. In this context, the careful selection of specific MRI slices that provide distinct anatomical details significantly enhances the ability to identify these early changes. The chief novelty of the study is that instead of selecting all slices, an approach for identifying the important slices is developed. The ADNI-3 dataset was used as the dataset when running the models for early detection of Alzheimer’s disease. Satisfactory results have been obtained by classifying with deep learning models, vision transformers (ViT) and by adding new structures to them, together with the model proposal. In the results obtained, while an accuracy of 99.45% was achieved with EfficientNetB2 + FPN in AD vs. LMCI classification from the slices selected with SSIM, an accuracy of 99.19% was achieved in AD vs. EMCI classification, in fact, the study significantly advances early detection by demonstrating improved diagnostic accuracy of the disease at the EMCI stage. The results obtained with these methods emphasize the importance of developing deep learning models with slice selection integrated with the Vision Transformers architecture. Focusing on accurate slice selection enables early detection of Alzheimer’s at the EMCI stage, allowing for timely interventions and preventive measures before the disease progresses to more advanced stages. This approach not only facilitates early and accurate diagnosis, but also lays the groundwork for timely intervention and treatment, offering hope for better patient outcomes in Alzheimer’s disease. The study is finally evaluated by a statistical significance test.

## Introduction

Alzheimer’s disease (AD) may be a common frame of dementia that causes memory misfortune and a common decay in cognitive work over time due to the passing of brain cells. At first, this disease manifests itself only through simple forgetfulness, begins to manifest itself in a more advanced manner over time. Within the progressed stages of the illness, assembly the patient’s fundamental needs and providing the necessary care can become an increasingly challenging and complex task.

Early detection of Alzheimer’s disease is valuable to prevent the rapid progression of the disease. Diagnosing the disease at an early stage allows the patient to carry out activities of daily living for longer. The application of diverse imaging modalities plays a crucial role in the diagnosis of the disease, and these techniques are intricately associated with the diagnostic process.

The disease is studied in different stages depending on its progression^1^. These stages generally range from healthy individuals to early mild cognitive impairment to advanced Alzheimer’s disease. In the Early Mild Cognitive Impairment (EMCI) stage, individuals may experience mild memory problems or other cognitive difficulties. In the Mild Cognitive Impairment (MCI) stage, they may experience cognitive difficulties such as forgetfulness or distraction. In the Late Mild Cognitive Impairment (LMCI) stage, they experience more severe memory problems, such as forgetting important events or information. They have more pronounced difficulties with activities of daily living. In the Alzheimer’s stage, there are severe cognitive problems such as memory loss, impaired decision-making, language and communication problems. Individuals become unable to carry out activities of daily living and may require full-time care. Radiological approaches used to diagnose AD include magnetic resonance imaging (MRI), computed tomography (CT), positron emission tomography (PET), functional MRI (fMRI), and single photon emission computed tomography (SPECT). Within the realm of MRI, various imaging techniques such as T1-weighted, T2-weighted, and proton-weighted images are utilized^2^.

Gaps in other literature studies have a major impact on the conduct of this research. Studies show that advanced-stage AD is relatively easy to detect, while the mild stage is more difficult to detect in AD^3^. At the same time, there is a lack of studies in the literature on how to select slices for the detection of Alzheimer’s disease in MR images. On the other hand, as a method, when determining the reference image for MRI slice selection, MRI slices were analyzed for each patient and the image with the highest number of edge segments among these slices was found and selected. Considering the techniques and methods used in this study, our motivation was the idea that the Feature Pyramid Network (FPN) structure integrated into the proposed model can improve diagnostic accuracy by extracting details more precisely. It is thought that integrating the methods and techniques used with an innovative approach can fill an important gap in the literature. Another main motivation for your work is to develop a model that can be used in clinical applications. Deep learning models such as Vision Transformers (ViT) and EfficientNet have been proven in the literature to help clinicians make earlier and more accurate decisions by making precise and reliable predictions from MRI images. This is thought to directly contribute to the quality of life of patients by facilitating early diagnosis in clinical practice. Based on this aim, the main motivation objectives of the study are as follows:

- To select only the necessary and meaningful slices instead of analyzing all slices. It would improve data quality and lead to more accurate analysis and results.
- To ensure that the disease is detected at the EMCI stage so that necessary precautions can be taken before progression to AD.
- Innovating deep learning models and providing a new model by integrating it with the Vision Transformers structure.

The article structure is as follows: We briefly introduce Alzheimer’s disease. Then, a literature review on AD is presented. Methodology is demonstrated following the dataset. In the methodology section, definitions of the models and evaluation metrics are given. Finally, the results and discussion along with future work are presented.

### Related work

Numerous considerations for determining Alzheimer’s disease are accessible within the literature. The following is a summary of a literature review of these studies.

In a recent study^4^, ran deep learning models for the diagnosis of Alzheimer’s disease with pre-trained networks and transfer learning using the ADNI dataset. They obtained the results by dividing the dataset into train and test. Data augmentation was performed by rotating the images in the dataset. The study received the results by running VGG-19, ResNet-50, and InceptionV3 models, yielding average accuracies of 97.54%, 97.16%, and 98.70%, respectively.

In a study^5^, proposed a model called Aux-ViT as an image transformation network architecture and solved some shallow feature problems with this proposal. Specifically, they added auxiliary multilayer sensors and chose ViT as the base network to eliminate prediction errors. They also utilized the ADNI-3 dataset, and an irregular manufactured cover based on pixel weighting combination to undertake information upgrade. They used T1-weighted and two-class dataset content, and split the training and test sets in an 8:2 ratio and reserved 20% of the training set for validation. They proposed online randomized engineered veil enlargement and multi-information combination upgrade to move forward MRIs. They also aimed to enhance multi-information fusion. Compared to the baseline ViT model, the Aux-ViT model achieved an accuracy of 89.58%. In their study, they presented a practical approach for early diagnosis of Alzheimer’s disease using MRI data.

In a study^6^, investigated and evaluated the applications of different CNN and transformer models on early detection of Alzheimer’s disease. They also presented a multimodal method for Alzheimer’s disease detection based on MRI and PET modality using a combination of EfficientNetV2 and a novel data augmentation and enhanced image transformer based on self-attention generative adversarial networks (SAGAN). They validated the proposed method using the Alzheimer’s Disease Neuroimaging Initiative (ADNI) and the Open Access Imaging Studies Series (OASIS). The proposed method achieved 96% accuracy by combining the key advantages of the image converter and EfficientNetV2.

In another study^7^, proposed TriFormer, a new transformer-based framework for classification using ADNI-1 and ADNI-2 datasets. They divided the dataset into 80% training and 20% testing and obtained the results with 50 epochs. They extracted multi-view picture highlights from MRI utilizing ViT. They obtained the results with a modality fusion transformer that combines the extracted multimodal features to perform more accurate transform predictions by combining image slices with a clinical class marker. They obtained an accuracy of 77.31% for the ADNI-1 dataset and 84.10% for the ADNI-2 dataset.

In a research^8^, conducted a classification analysis of T1-weighted MRI images utilizing the ADNI dataset. They proposed a new model which is a hybrid three-dimensional CNN and transformer design. In addition to the ADNI dataset, they also tested the same model on OASIS and AIBL datasets. They compared this model with eight basic algorithms. The dataset was partitioned such that 80% was allocated for training purposes, while the remaining 20% was designated for testing. Their proposed LongFormer model achieved 93.43% accuracy on the ADNI dataset.

In a research^9^, conducted a study to develop a new model for computer-aided diagnosis (CAD). In their study, they performed data alignment and merging using the ADNI dataset. They applied a method called AliFuse for aligning and merging data from different modalities. This model aims to integrate information from different modalities by processing data from different modalities. The data sets were partitioned into three segments: 70% for training, 20% for testing, and 10% for validation purposes. The proposed model achieved an average accuracy of 87.93% for the three classes.

In a different study^10^, conducted a classification task using the ADNI dataset. They augmented the dataset with rotation operations. They used 1.5T and 3 T weighted three-dimensional images, and obtained the results through 5-fold cross validation. A hybrid (ensemble) model by combining ViT and CNN model was utilized yielding 89.46% accuracy for CN vs. AD, 78.60% accuracy for MCI vs. AD and 78.86% accuracy for CN vs. MCI.

In a study^11^, used the ADNI-3 dataset for the classification of Alzheimer’s disease and divided the dataset into 70% train and 30% test. There are three classes in the data sets. They obtained an accuracy of 98.94% for CN vs. AD, 97.95% for MCI vs. AD and 98.42% for CN vs. MCI classification with EfficientNetB0 model.

**Table 1:** Summary of literature Studies.

Reference	Year	Dataset	Models	Class	Accuracy
Cholli & Naveen	2024	ADNI	VGG-19 ResNet-50 InceptionV3	CN EMCI MCI LMCI AD	97.54% 97.16% 98.70%
Duan et al.	2023	ADNI-3	Aux-ViT	AD vs. CN	89.58%
Kadri et al.	2022	ADNI OASIS	DenseNet121 Proposed ViT	AD, CN	86.00% 96.00%
Liu et al.	2023	ADNI-1 ADNI-2	TriFormer (ViT)	AD, MCI, CN	77.31% 84.10%
Chen et al.	2024	ADNI	LongFormer (ViT)	AD, MCI, CN	93.43%
Chen et al.	2024	ADNI	AliFuse (ViT)	AD, MCI, CN	87.93%
Gamal et al.	2022	ADNI	CNN + ViT (ensemble)	AD vs. CN AD vs. MCI MCI vs. CN	89.46% 78.60% 78.86%
Şener et al.	2024	ADNI-3	EfficientNetB0 EfficientNetB0 DenseNet121	CN vs. AD MCI vs. AD CN vs. MCI	98.94% 97.95% 98.42%

An outline of the related studies is given in Table 1. To summarize briefly, the studies reviewed in this paper have achieved their results by using profound learning methods and Vision Transformers structures. In the subsequent sections, we introduce advanced deep learning models that utilize a more contemporary ADNI dataset, alongside our novel model and the methodology employed for selecting the slice.

## Materials & methods

In this study, images including axial brain slices and T1-weighted structural MRI data were used to diagnose Alzheimer’s disease. EfficientNetB2, InceptionV3, Regnetx006 and the proposed new models were employed on 224 × 224 rescaled MRI images for early-stage diagnosis of Alzheimer’s disease. While preparing the dataset, 10-fold cross validation was utilized. K-fold cross validation includes partitioning the information into K diverse subsets. At each emphasis, one subset is utilized for testing, whereas the remaining K-1 subsets are utilized for training. The validity of the model is determined by the average accuracy obtained as a result of these K iterations^12^. The hyperparameters used in the model are given in Table 2.

**Table 2:** Properties of the hyperparameters used in the model.

HyperParameters	Value
Optimization algorithm	Adam
Learning Rate	0.001
Epoch	10
Batch Size	16

**Fig. 1:**
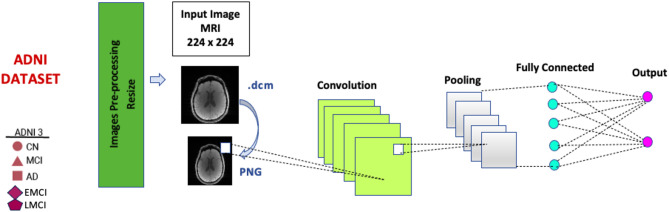
The structure of a general CNN architecture.

Deep learning models were applied for early detection of Alzheimer’s disease on a total of 24,661 neuroimaging (MRI) data in the dataset to facilitate the processing of DICOM image files in the preprocessing stage, the images with the.dcm extension were transformed into PNG format. Google Colab was used as the infrastructure instead of the operating system. The training of the models was conducted within the Google Colab environment, utilizing TensorFlow, Python version 3.7, and the Scikit-learn library. A100 GPU and NDVIA graphics processing unit (GPU) were used in the experiments and the results were generated through Google Colab platform. The A100 GPU offers a choice of 40 GB or 80 GB HBM2e (High Bandwidth Memory) memory capacity. Results were obtained using 80 GB memory capacity. This large memory is sufficient for processing large AI models or datasets. As for the processor, the A100 GPU contains a total of 6,912 Compute Unified Device Architecture (CUDA) cores. CUDA cores accelerate graphics processing as well as general-purpose computing and provide high performance for parallel processing. Following the conversion of the images to PNG format, they were matched with the corresponding class labels present in the CSV file. After the labeling process was completed, the selected current models were run. Stratified 10-fold cross validation was used to separate the training and test dataset to solve the imbalance problem that occurs when the ratio of data between classes in the dataset is not equal. Figure-1 shows the structure of a general CNN architecture.

### Dataset

ADNI-3 dataset obtained from the ADNI database (available at http://adni.loni.usc.edu). Launched in 2016, the ADNI-3 dataset aims to describe in detail the associations between genetic, clinical, cognitive, imaging and biochemical biomarkers across the spectrum of Alzheimer’s disease. ADNI-3 moreover incorporates checks that distinguish tangles of tau proteins (tau PET), a key pointer of the infection^13^. We used the ADNI-3 dataset for the analyses as we found that the ADNI-3 dataset contains the most up-to-date data.

In this study, we used MRI images from the ADNI-3 dataset of 627 individuals, including 50 AD, 37 EMCI, 160 MCI, 15 LMCI and 365 CN patients. The dataset includes 341 female and 286 male subjects. Each individual has 54 slices of MRI images. And there are also extra MRI images of a patient taken in subsequent years. The dataset contains the last updated data added in April 2024. Pyhton programming language was used to perform the analyses.

AD refers to individuals with Alzheimer disease, CN refers to healthy individuals, and MCI refers to individuals with mild Alzheimer’s disease. EMCI refers to an earlier stage of cognitive impairment, while LMCI refers to a more advanced stage of cognitive impairment. EMCI is usually characterized by less prominent symptoms and therefore early diagnosis can be more difficult. LMCI is recognized as a stage with a higher risk of developing into Alzheimer’s disease. Table 3 presents the classes, the number of subjects and the total number of scans per class.

**Table 3:** Individuals and total number of scans.

Class	Individuals	Total Scans
CN	365	19,926
EMCI	37	4253
MCI	160	10,285
LMCI	15	1826
AD	50	6380

The images in the dataset consist of images with.dcm file extension. Portable network graphics (PNG) images were obtained by converting DICOM images. During the data preprocessing phase, each MRI image fed into our convolutional neural network (CNN) model was resized to dimensions of 224 × 224 pixels, in accordance with the requirements of the model architectures that necessitate input images of this specific size.

### Structural similarity index measure (SSIM)

It is a metric used to measure the similarity between two images. SSIM is used to evaluate the quality of images by trying to mimic human visual perception. It is widely used in image compression, noise removal, image reconstruction and image classification. SSIM takes into account three main components: the average brightness values, brightness variation and structural similarity of the two images. The SSIM value varies between − 1 and 1. 1 indicates that the two images are exactly the same, while 0 indicates that they are completely different. When SSIM is −1, this means that the two images have completely opposite structural properties. Since SSIM is a purely mathematical calculation, the results are always consistent and not affected by subjective judgments. This eliminates the possibility of different people making different judgments on the same image. The manual selection process can be time-consuming and tedious when working with large datasets. Using SSIM, similarity can be measured automatically, speeding up the process. SSIM is able to accurately detect even small structural differences between images. This ensures that the small details that need to be analyzed in MRI images are not missed. Furthermore, SSIM’s proximity to human visual perception helps to achieve more meaningful and accurate results in the evaluation of MRI images^14^.

In the present study, we used SSIM to select the correct slices in Alzheimer’s disease. While obtaining the results, 5 slices were manually selected for comparison (slices 25, 26, 27, 28 and 29). Since the study^15^, emphasized the importance of the middle slices in MRI images for Alzheimer’s disease, the middle slices in MRI images containing 54 slices were preferred for manual selection. Given a reference image, to see whether SSIM selection works correctly, we obtained SSIM scores for both informative (closer to the center slice) and non-informative (far from the center slice) MRI images, and observed consistent results. For slice selection based on SSIM, the first step was to determine a reference image for each class. When determining the reference image, the MRI slices for each patient were analyzed and the image with the largest number of edge segments among these slices was found and selected. When finding the reference image for each class, a reference image was determined for each patient and finally a generic reference image with the largest number of edge segments was selected from the reference images. To determine the necessary slices, we first selected a reference image for each patient. Then, we chose another reference image by looking at the reference images from all patients within the same class. To clarify how the edge segments were identified in the reference image selection process, we utilized a Canny edge detector rather than simple size-based criteria. The slice with the highest number of edges was selected as the subject-level reference image. This procedure was repeated across all subjects in a class, and the class-level reference image was chosen as the one with the maximum edge count among them. This method allowed us to objectively identify slices with the richest anatomical information, which is critical for accurate SSIM-based similarity scoring and reliable slice selection. To identify the reference slice with the richest anatomical content for each subject, we first applied the Canny edge detection algorithm to each axial slice of the brain MRI volume. The Canny method was selected for its robustness in detecting meaningful anatomical boundaries while minimizing noise-related artifacts. To identify the reference slice with the richest anatomical content for each subject, we first applied the Canny edge detection algorithm to each axial slice of the brain MRI volume. The Canny method was chosen due to its strong performance in detecting meaningful anatomical contours while suppressing noise. For each slice, a binary edge map E ∈ {0, 1}^{H × W}was generated, where E(i, j) = 1 indicates the presence of an edge at pixel location (i, j). The edge density score of a slice is calculated as given in Eq. (1):

1 $$\:\:\:\:\:\:\:\:\:\:\:\:\:\:\:\:\:\:\:\:\:\:\:\:\:\:\:\:\:\:\:\:\:\:\:\:\:\:\:\:\:\:\:\:\:\:\:\:\:\:\:\:\:\:\:\:\:\:\:\:\:\:\:\:\:\:\:\:\:\:\:\:\:\hspace{1em}S\:=\sum\:_ {\rm i=1}^{\rm H}\:\:\sum\:_{\rm j=1}^{\rm W}{\rm {E}({i},\:{j})}$$

where SS represents the total number of edge pixels in the slice. The slice with the maximum edge pixel count was selected as the subject-level reference image. This process was repeated for each subject in the class, and the class-level reference image was selected as the one with the highest edge pixel count among all subject-level reference slices.

The Structural Similarity Index (SSIM) is a perceptual metric that quantifies the similarity between two images. Unlike traditional metrics like Mean Squared Error (MSE), which focus only on pixel differences, SSIM evaluates the structural information in an image by considering three main components^16^:

- Luminance (L) – Measures the difference in brightness between the two images.
- Contrast (C) – Evaluates the difference in contrast between the images.
- Structure (S) – Analyzes the correlation of pixel patterns between the images.

The SSIM formula is given by Eq. (2):

2 $$\:\:\:\:\:\:\:\:\:\:\:\:\:\:\:\:\:\:\:\:\:\:\:\:\:\:\:\:\:\:\:\:\:\:\:\:\:\:\:\:\:\:\:\:\:\:\:\:\:\:\:\:\:\:\:\:\:\:\:\:\:\:\:\:\:\:\:\:\:\:\:\:\:\:\:\:\:\:SSIM\:(x,y)=\frac{(\text{}{2}_{{\upmu\:}x{\upmu\:}y}+\text{C}1\text{})({2{\upsigma\:}}_{xy}\text{}+\text{C}2\text{})}{\left({{\upmu\:}}^{2}x+{{\upmu\:}}^{2}y+C1\right)({{\upsigma\:}}^{2}x+{{\upsigma\:}}^{2}y\text{}+\text{C}2\text{})}$$

Where x and y are the two images being compared. The terms µx​ and µy​ represent the mean intensity values of images x and y, respectively. The variances of these images are denoted as $\:{{\upsigma\:}}^{2}x$ ​ and $\:{{\upsigma\:}}^{2}y\text{}$​ and quantify their contrast. Additionally, $\:{{\upsigma\:}}_{xy}\:$represents the covariance between the two images, capturing their structural similarity. To prevent division by zero and stabilize the computation, small constants C1​ and C2​ are introduced in the formula.

**Fig. 2:**
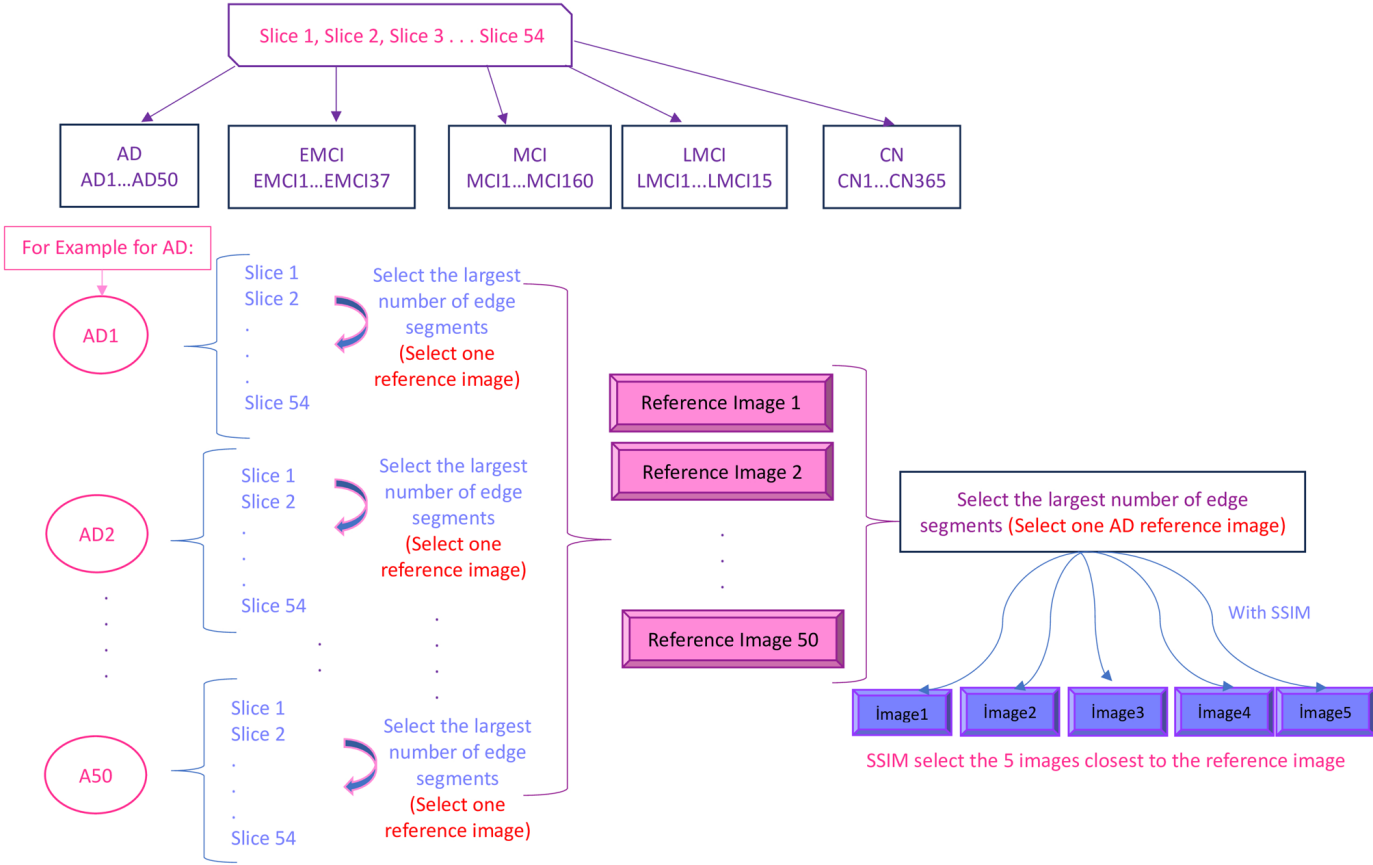
Reference image selection with SSIM.

As mentioned above, Figure-2 illustrates the SSIM-based slice selection process. In summary, the slice with the highest number of edge segments among the 54 MRI slices was selected as the patient-level reference image for each patient. Then, the reference image with the most edge segments across all patients in the same class was chosen as the class-level reference image. SSIM was then used to compare each slice with the reference image, allowing the identification of slices most structurally similar to the reference. This ensured that the selected slices were informative and representative, focusing on anatomical consistency relevant to Alzheimer’s disease.

**Fig. 3:**
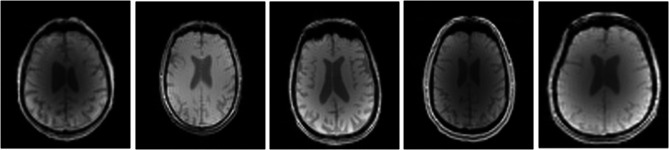
Selected reference images for AD, EMCI, MCI, LMCI and CN classes, respectively.

In this selection process, we prioritized the images with the largest number of edge segments to choose the final reference image again. The slices of the reference images for each class selected with SSIM were as follows. Slice #30 for CN and EMCI, #31 for MCI, #33 for LMCI and AD classes were selected as reference images, illustrated in Figure-3. These reference images selected for each class were used to select 5 images with SSIM. SSIM would improve data quality and lead to more accurate analysis and results.

### Performance metrics

Different metrics are used in models when evaluating their performance. The accuracy metric appears the extent of accurately classified tests and gives a diagram of the quality of the expectation. Precision measures how many of the occurrences that the model classifies as positive are actually positive. Recall measures the extent of actual positive cases that the model classifies as positive. The F1-score measures the performance of the model in terms of both accuracy and precision, while balancing these two metrics. For this reason, it is often the metric of choice for performance evaluations^17^. Area Under the Curve (AUC) usually refers to the area under the ROC (Receiver Operating Characteristic) curve and is a metric used to evaluate the performance of classification models. The ROC curve graphically shows the accuracy, sensitivity and specificity of the model at different thresholds. AUC measures the performance of a classification model with a single numerical value. The AUC value ranges from 0 to 1, indicating that the model performs excellently as it approaches higher values^18^. The Matthews Correlation Coefficient (MCC) is a metric that ranges from − 1 to + 1, reflecting the quality of binary classification predictions. A value of + 1 signifies a perfect prediction where the classifier correctly identifies all positive and negative cases. An MCC of 0 indicates that the prediction performance is no better than random guessing, meaning the classifier has no real predictive power. Conversely, a value of −1 represents a complete disagreement between the predicted and actual classes, indicating that the model is consistently wrong in its predictions.

These metrics are calculated using Eqs. (3)-(7). The components of the equations are true positive (TP), true negative (TN), false positive (FP) and false negative (FN).

3 $$\:\varvec{A}\varvec{c}\varvec{c}\varvec{u}\varvec{r}\varvec{a}\varvec{c}\varvec{y}=\frac{\varvec{T}\varvec{P}+\varvec{T}\varvec{N}\:\:\:\:\:\:\:\:\:}{\varvec{T}\varvec{P}+\varvec{T}\varvec{N}+\varvec{F}\varvec{P}+\varvec{F}\varvec{N}}\:\:$$

4 $$\:\varvec{P}\varvec{r}\varvec{e}\varvec{c}\varvec{i}\varvec{s}\varvec{i}\varvec{o}\varvec{n}=\:\frac{\varvec{T}\varvec{P}}{\varvec{T}\varvec{P}+\varvec{F}\varvec{P}}\:$$

5 $$\:\varvec{R}\varvec{e}\varvec{c}\varvec{a}\varvec{l}\varvec{l}=\:\frac{\varvec{T}\varvec{P}}{\varvec{T}\varvec{P}+\varvec{F}\varvec{N}}\:\:$$

6 $$\:\varvec{F}1-\varvec{s}\varvec{c}\varvec{o}\varvec{r}\varvec{e}=2\varvec{*}\:\frac{\varvec{P}\varvec{r}\varvec{e}\varvec{c}\varvec{i}\varvec{s}\varvec{i}\varvec{o}\varvec{n}\varvec{*}\varvec{R}\varvec{e}\varvec{c}\varvec{a}\varvec{l}\varvec{l}}{\varvec{P}\varvec{r}\varvec{e}\varvec{c}\varvec{i}\varvec{s}\varvec{i}\varvec{o}\varvec{n}+\varvec{R}\varvec{e}\varvec{c}\varvec{a}\varvec{l}\varvec{l}}\:$$

7 $$\:\varvec{M}\varvec{C}\varvec{C}=\:\frac{\left(\varvec{T}\varvec{P}\varvec{*}\varvec{T}\varvec{N}\right)-\left(\varvec{F}\varvec{P}\varvec{*}\varvec{F}\varvec{N}\right)}{\sqrt{(\varvec{T}\varvec{P}+\varvec{F}\varvec{P})(\varvec{T}\varvec{P}+\varvec{F}\varvec{N})(\varvec{T}\varvec{N}+\varvec{F}\varvec{P})(\varvec{T}\varvec{N}+\varvec{F}\varvec{N})}}$$

The experiments conducted in this research utilized the A100 GPU, with results generated through the Google Colab platform. These graphics processing units provide a significant benefit in computationally intensive tasks, such as deep learning, which fundamentally enhances performance and efficiency.

### McNemar’s test

McNemar’s Test allows to evaluate the difference between two models without the assumption.

of independence. It is especially used in binary classification problems. McNemar’s Test is a reliable method for determining whether one model has a significant advantage over the other. As it is a test of statistical significance, it is used to determine whether the difference between models is due to chance^19^.

### Models

EfficientNetB2, InceptionV3, RegNetx006, and a basic Vision Transformer structure (ViT) were used as models. In addition, a new model was tested by adding a Feature Pyramid Network (FPN) structure to EfficientNetB2. The FPN combines feature maps of different resolutions extracted by deep neural networks, enabling better recognition of objects at various scales. The main goal of FPN is to effectively combine multi-scale information from different layers, combining high- and low-level features. It involves an upward progression starting from the top-level feature map, and at each step the top-level feature map is transformed into a higher resolution map by upsampling^20^. In this process, the feature map of the previous level and the map of the lower level are merged. In this proposed model, feature maps of different resolutions are obtained by outputting certain layers from the EfficientNetB2 model. These layers obtain the feature maps extracted from the image depending on the depth of the model. In FPN, these multi-resolution feature maps from EfficientNetB2 are combined by upsampling and addition. FPN was added to EfficientNet because it is often preferred when working with complex and multi-layered data structures such as medical image analysis. FPN is considered to have the following advantages on Alzheimer’s detection:

- Brain images often contain both large-scale and small-scale changes. By extracting these features at different resolutions, FPN helps the model learn both global and local information.
- Small changes in brain tissue can be important in the early diagnosis of Alzheimer’s disease. By using high-resolution feature maps, FPN can help capture these fine details and minimize errors in diagnosis.
- FPN provides benefits in classification such as better feature representation, resolution independence and rich feature hierarchy.
- FPN can better capture and detect anomalies or changes at various resolutions, which can help catch early signs of Alzheimer’s disease.

**Fig. 4:**
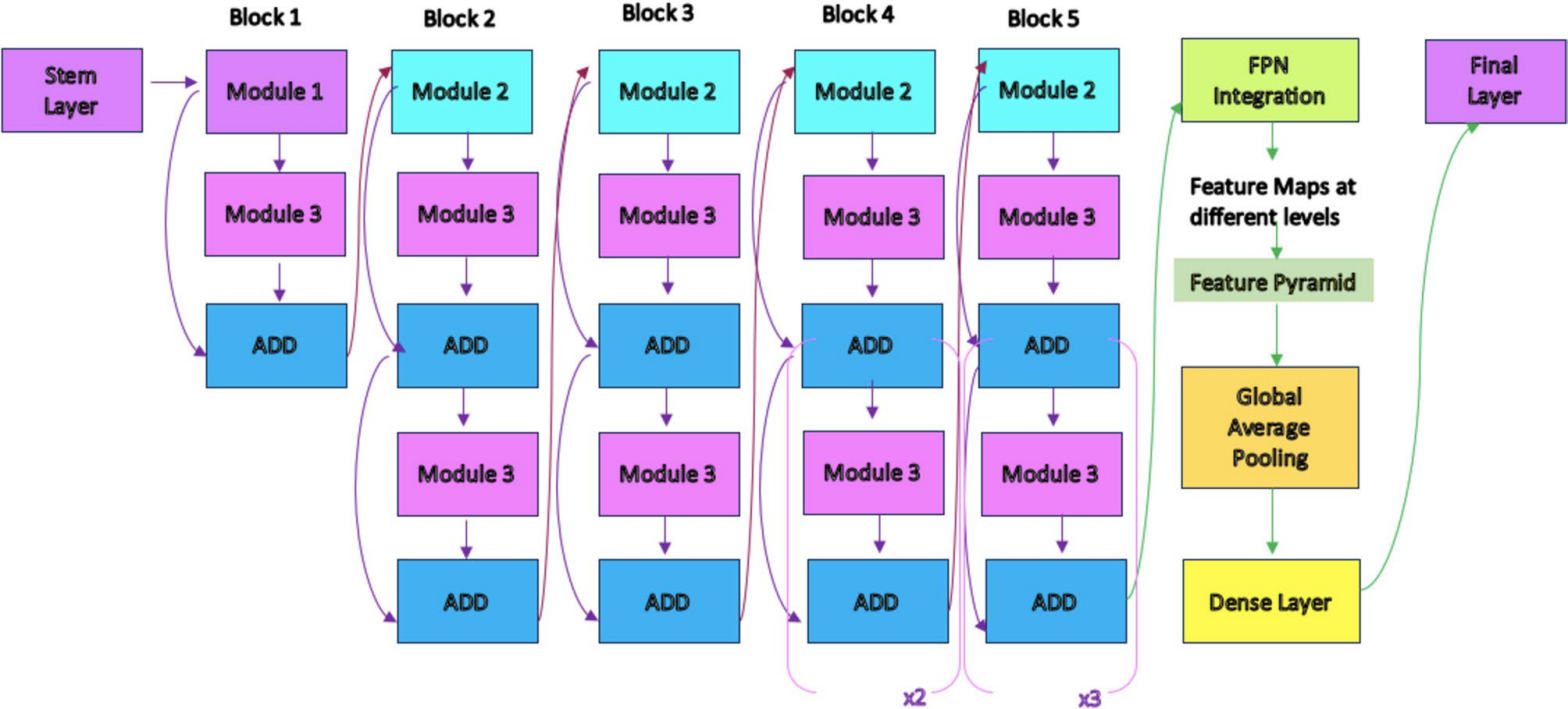
EfficientNetB2 + FPN architecture.

Figure 4 shows a visualization of this EfficientNetB2 + FPN structure.

**Fig. 5:**
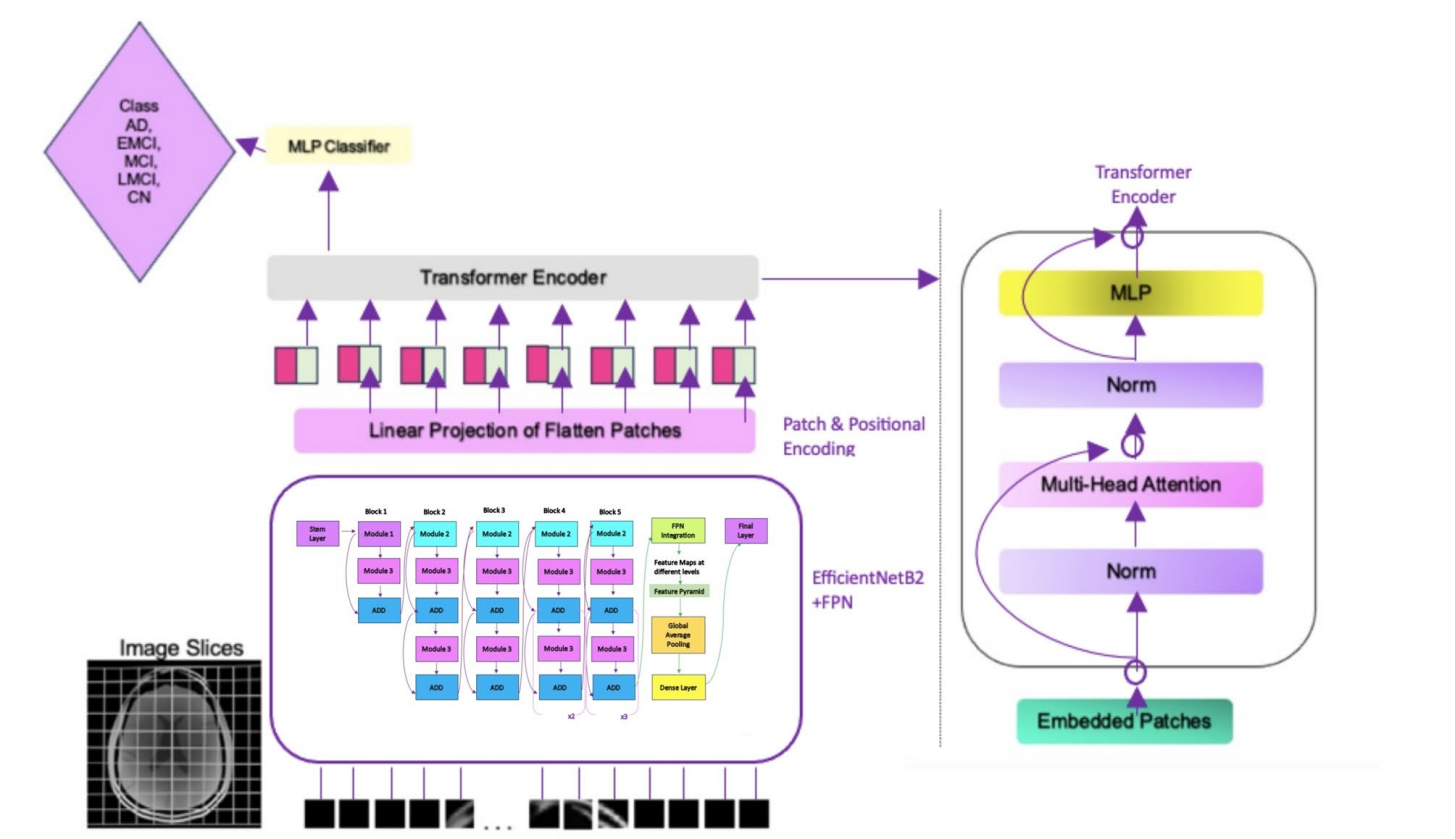
The proposed ViT + EfficientNetB2 + FPN architecture.

Apart from the new model described above, a new model was created by integrating the ViT structure with the EfficientNetB2 + FPN structure. The architecture of this model is shown in Fig. 5. The image is divided into squares of a given size (e.g. 16 × 16) or small pieces called “patches”. Each patch is converted into a flat vector. In our Vision Transformer architecture, we employ a transformer encoder consisting of 8 layers, each with eight attention heads and an embedding dimension of 768. The model follows the standard ViT formulation, with multi-head self-attention and feed-forward sublayers in each encoder block. To integrate multi-scale features from the FPN into the Vision Transformer, each FPN output (P3–P5) is first projected to a standard embedding dimension via 1 × 1 convolutions, resized to a uniform spatial resolution, and then concatenated along the channel axis before being flattened into patch embeddings compatible with the ViT input format. After that it is sent to the EfficientNetB2 + FPN structure. The feature maps coming from the EfficientNetB2 + FPN structure are adapted to ViT and then processed within ViT. In this way, the power of CNN-based rich feature maps is combined with the global context learning capabilities of ViT.

The multi-scale information extraction capability of EfficientNetB2 + FPN, combined with ViT’s capacity to learn strong spatial relationships, the classification performance has been improved. The parameter efficiency of EfficientNetB2, the multi-scale integration of FPN and the overall model complexity of ViT can reduce the risk of model overfitting. The proposed new model is expected to provide a faster and more efficient training process, allowing researchers to obtain results in a shorter time. In our model, intermediate feature maps from different stages of EfficientNetB2 are used to feed the Feature Pyramid Network (FPN). Specifically, the outputs of Block 4 (P3 level), Block 6 (P4 level), and Block 8 (P5 level) are utilized. These multi-scale features enable the FPN to learn more meaningful and detailed representations.

## Results and discussions

In this study, neuroimaging (MRI) data consisting of five classes (CN, EMCI, MCI, LMCI and AD) were used. Three different CNN models and a simple ViT model were tested for early detection of AD. In addition, two new models were proposed. The training of the models was performed on Google Colab.

Once the studied images were converted to PNG, the images in the dataset were matched with labels of each class in the CSV file. After labeling process was completed, the selected models (EfficientNetB2, InceptionV3, RegNetx006) were run with 10 epochs and 10-fold cross validation. To avoid overfitting and improve the performance of the models, optimizing the CNN models with a dropout process is an important step. The results were first obtained based on 5 manually selected slices and then 5 other slices automatically selected with SSIM. A reference brain MRI image was given for each class when selecting slices with SSIM. The results are given in Tables 4 and 5.

**Table 4:** Classification results obtained when the models are run (5 slices manually).

Models	Accuracy	Precision	Recall	F1 Score	AUC	MCC
EfficientNetB2	0.9177	0.8244	0.7478	0.7834	0.9569	0.7823
InceptionV3	0.8788	0.7148	0.6556	0.6864	0.9381	0.6972
RegNetx006	**0.9712**	0.9345	0.9203	0.9268	0.9929	0.9375
Basic ViT	0.8898	0.7678	0.6981	0.7302	0.9482	0.7394
EfficientNetB2 + FPN (Our Model)	0.9473	0.9315	0.9029	0.9167	0.9714	0.9098
ViT + EfficientNetB2 + FPN (Our model)	0.9091	0.9082	0.7089	0.7978	0.9021	0.8236

**Table 5:** Classification results obtained when the models are run (5 slices with SSIM).

Models	Accuracy	Precision	Recall	F1 Score	AUC	MCC
EfficientNetB2	0.9688	0.9312	0.9110	0.9178	0.9886	0.9125
InceptionV3	0.8875	0.7533	0.6508	0.6995	0.9345	0.6912
RegNetx006	**0.9889**	0.9733	0.9713	0.9701	0.9981	0.9672
Basic ViT	0.9362	0.8487	0.8288	0.8390	0.9777	0.8354
EfficientNetB2 + FPN (Our Model)	0.9874	0.9734	0.9633	0.9671	0.9974	0.9610
ViT + EfficientNetB2 + FPN (Our model)	0.9517	0.8593	0.7585	0.8065	0.9078	0.8041

Looking at the average accuracy values obtained in Tables 4 and 5, it can be said that the best results are obtained with the RegNetx006 model in both manual and SSIM-based scenarios. It is also observed that the slices selected with SSIM obtained better results compared to the slices selected manually. At this point, we can see the importance of the proposed slice selection mechanism with SSIM. It is seen that our proposed EfficientNetB2 + FPN model gives quite satisfactory results, coming after RegNetx006. In the second scenario with automated slice selection, RegNetx006 and EfficientNet + FPN models yield 98.89% and 98.74% accuracies, respectively.

**Fig. 6:**
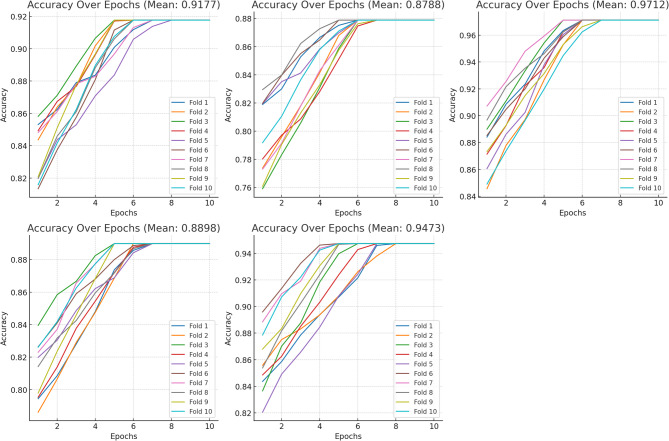
Manually selected results (**A**) EfficientNetB2, (**B**) InceptionV3, (**C**) RegNetx006, (**D**) Basic ViT (**E**) EfficientNetB2 + FPN.

**Fig. 7:**
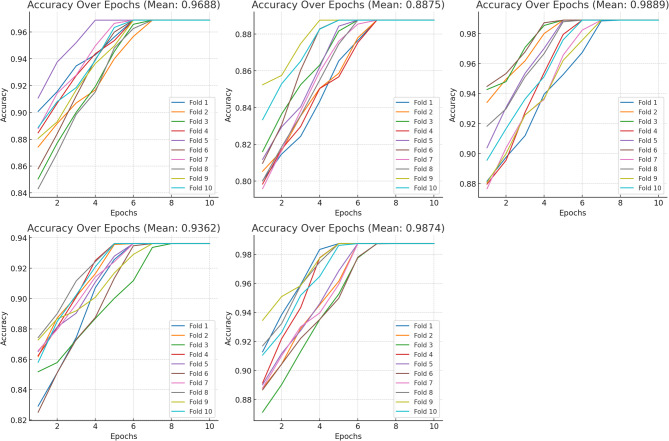
With SSIM selected results (**A**) EfficientNetB2, (**B**) InceptionV3, (**C**) RegNetx006, (**D**) Basic ViT (**E**) EfficientNetB2 + FPN.

The graphs showing the average accuracy values for each model and the accuracy values at each epoch are given in Figs. 6 and 7.

The models were also run with 1 vs. 1 classification, where each class was compared with another. The purpose of 1 vs. 1 classification is to make a direct comparison of the two models, to clearly identify which methods perform better. Table 6 (5 slices manually) and Table 7 (5 slices with SSIM) show these results.

When we investigate Table 6, the best accuracy of 99.12% is obtained with the EfficinetNetB2 + FPN model in the AD vs. LMCI comparison. This is followed by 98.94% with the RegNetx006 model in the MCI vs. LMCI comparison. Achieving good results in the AD vs. LMCI comparison is critical in terms of detecting the patient from the LMCI stage to the AD stage. In the MCI vs. LMCI comparison, it is important to detect the patient before the patient progresses to advanced MCI and AD.

When we look at Table 7, the best accuracy result of 99.45% is obtained with the EfficinetNetB2 + FPN model in the AD vs. LMCI comparison. This is followed by 99.42% for the RegNetx006 model in the AD vs. LMCI comparison. Achieving good results in the AD vs. LMCI comparison is also an important issue as mentioned in Table 6. In AD vs. EMCI classification, EfficientNetB2 + FPN achieved 99.19% accuracy. Likewise, in the AD vs. EMCI comparison, it is thought that the patient having an early MCI would have a significant impact on the ability to detect AD at an early stage when it is detected before the AD stage. Apart from these, when manually selected MRI images are compared with the images chosen with SSIM, it can be said that there are considerable improvements in the results obtained from the images chosen with SSIM, which stresses the importance of the automated selection mechanism.

The Matthews Correlation Coefficient (MCC) values presented in Table 7 demonstrate the robustness and reliability of the proposed models across all pairwise classification tasks. Notably, the models incorporating the EfficientNetB2 backbone combined with the Feature Pyramid Network (FPN), as well as the integrated ViT + EfficientNetB2 + FPN model, consistently achieve higher MCC scores compared to baseline architectures such as EfficientNetB2, InceptionV3, RegNetx006, and Basic ViT. For instance, in challenging classifications such as CN/AD and CN/MCI, the MCC values for the combined models reach above 0.94, indicating strong agreement between predicted and true labels. Furthermore, even in subtler distinctions like MCI/EMCI and LMCI/EMCI, these models maintain MCC scores above 0.83, reflecting their effectiveness in capturing nuanced patterns within the data. Overall, the elevated MCC metrics across diverse classification pairs highlight the superiority of the proposed multi-scale and transformer-enhanced architectures in delivering balanced and reliable predictions, addressing potential class imbalance and enhancing overall model performance.

In this study, McNemar’s test was used, the nonparametric version of the χ2 test, to assess the statistical significance between the performances of the classifiers. Comparing two classifiers results in four possible outcomes, shown in Table 10.

As outlined in Table 10, Nff, Nsf, Nfs, and Nss correspond to the cases where both classifiers failed, only classifier A was correct, only classifier B was correct, and both classifiers were correct, respectively. However, only Nsf and Nfs were considered to identify a significant difference, as they reflect the instances where one classifier succeeded while the other did not. These values were used to calculate the z-score, which determines whether the two classifiers perform similarly, as shown in Eq. (8).

8 $$\:\varvec{Z}=\frac{(\left|{\varvec{N}}_{\varvec{s}\varvec{f}}-{\varvec{N}}_{\varvec{f}\varvec{s}}\right|-1)}{\sqrt{{\varvec{N}}_{\varvec{s}\varvec{f}}}\:+{\varvec{N}}_{\varvec{f}\varvec{s}}}$$

When the z-score is 0, it indicates that the two classifiers have similar performance. As the z-score moves further from 0, the difference in performance between the two classifiers becomes more pronounced. Moreover, z-scores can be analyzed based on confidence levels for both one-tailed and two-tailed tests. The confidence levels associated with various z-scores are shown in Table 11.

**Table 6:** Classification results obtained when the models are run 1 vs. 1 (5 slices manually).

1 vs. 1	Models	Accuracy	Precision	Recall	F1 Score	AUC	MCC
CN/AD	EfficientNetB2 InceptionV3 RegNetx006 Basic ViT EfficientNetB2 + FPN (Our Model) ViT + EfficientNetB2 + FPN (Our Model)	0.9756 0.9012 0.9858 0.9128 **0.9881** 0.9859	0.9473 0.7627 0.9673 0.7922 0.9718 0.9645	0.9423 0.8194 0.9612 0.7582 0.9688 0.9640	0.9446 0.7927 0.9670 0.7757 0.9720 0.9646	0.9838 0.9719 0.9882 0.9591 0.9982 0.9861	0.9491 0.8375 0.9702 0.8236 0.9734 0.9668
CN/MCI	EfficientNetB2 InceptionV3 RegNetx006 Basic ViT EfficientNetB2 + FPN (Our Model) ViT + EfficientNetB2 + FPN (Our Model)	0.9649 0.9283 **0.9891** 0.9172 0.9872 0.9788	0.9448 0.8289 0.9718 0.7983 0.9696 0.9469	0.9429 0.8194 0.9799 0.7681 0.9664 0.9469	0.9440 0.8267 0.9731 0.7824 0.9685 0.9469	0.9819 0.9519 0.9995 0.9675 0.9982 0.9711	0.9474 0.8299 0.9754 0.7903 0.9698 0.9469
CN/LMCI	EfficientNetB2 InceptionV3 RegNetx006 Basic ViT EfficientNetB2 + FPN (Our Model) ViT + EfficientNetB2 + FPN (Our Model)	0.9783 0.9192 0.9896 0.9189 0.9881 **0.9898**	0.9492 0.8122 0.9746 0.7997 0.9715 0.9797	0.9413 0.7895 0.9735 0.7817 0.9691 0.9794	0.9445 0.8019 0.9742 0.7906 0.9745 0.9795	0.9962 0.9691 0.9987 0.9781 0.9818 0.9822	0.9507 0.8008 0.9741 0.7923 0.9717 0.9795
CN/EMCI	EfficientNetB2 InceptionV3 RegNetx006 Basic ViT EfficientNetB2 + FPN (Our Model) ViT + EfficientNetB2 + FPN (Our Model)	0.9769 0.8983 0.9883 0.9247 0.9850 **0.9892**	0.9428 0.7712 0.9718 0.8283 0.9650 0.9728	0.9438 0.7912 0.9683 0.8012 0.9600 0.9382	0.9427 0.7812 0.9742 0.8140 0.9628 0.9548	0.9849 0.9591 0.9819 0.9699 0.9875 0.9884	0.9431 0.7806 0.9714 0.8144 0.9626 0.9529
AD/MCI	EfficientNetB2 InceptionV3 RegNetx006 Basic ViT EfficientNetB2 + FPN (Our Model) ViT + EfficientNetB2 + FPN (Our Model)	0.9819 0.9383 0.9881 0.9239 **0.9900** 0.9890	0.9592 0.8619 0.9891 0.8634 0.9864 0.9508	0.9521 0.8371 0.9721 0.7898 0.9737 0.9491	0.9540 0.8492 0.9795 0.8276 0.9798 0.9502	0.9920 0.9781 0.9961 0.9695 0.9986 0.9818	0.9567 0.8494 0.9820 0.8269 0.9800 0.9500
AD/LMCI	EfficientNetB2 InceptionV3 RegNetx006 Basic ViT EfficientNetB2 + FPN (Our Model) ViT + EfficientNetB2 + FPN (Our Model)	0.9449 0.9693 0.9942 0.9757 **0.9945** 0.9697	0.8544 0.9382 0.9809 0.9369 0.9868 0.9268	0.8529 0.9193 0.9891 0.9371 0.9856 0.9215	0.8515 0.9283 0.9799 0.9360 0.9850 0.9226	0.9733 0.9955 0.9973 0.9947 0.9988 0.9503	0.8529 0.9274 0.9850 0.9367 0.9861 0.9236
AD/EMCI	EfficientNetB2 InceptionV3 RegNetx006 Basic ViT EfficientNetB2 + FPN (Our Model) ViT + EfficientNetB2 + FPN (Our Model)	0.9895 0.9482 0.9822 0.9683 **0.9919** 0.9475	0.9742 0.8910 0.9810 0.9099 0.9813 0.8741	0.9739 0.8618 0.9701 0.9074 0.9780 0.8614	0.9740 0.8783 0.9748 0.9072 0.9791 0.8687	0.9917 0.9891 0.9946 0.9889 0.9988 0.9261	0.9739 0.8763 0.9753 0.9082 0.9791 0.8671
MCI/LMCI	EfficientNetB2 InceptionV3 RegNetx006 Basic ViT EfficientNetB2 + FPN (Our Model) ViT + EfficientNetB2 + FPN (Our Model)	0.9518 0.8973 0.9881 0.9181 **0.9891** 0.9887	0.9129 0.7672 0.9784 0.7896 0.9737 0.9621	0.9192 0.7221 0.9794 0.7724 0.9717 0.9612	0.9172 0.7435 0.9782 0.7797 0.9701 0.9626	0.9839 0.9719 0.9889 0.9691 0.9985 0.9808	0.9164 0.7442 0.9786 0.7806 0.9718 0.9619
MCI/EMCI	EfficientNetB2 InceptionV3 RegNetx006 Basic ViT EfficientNetB2 + FPN (Our Model) ViT + EfficientNetB2 + FPN (Our Model)	0.9749 0.8771 0.9795 0.9164 **0.9888** 0.9862	0.9317 0.7219 0.9484 0.8091 0.9739 0.8496	0.9311 0.6329 0.9463 0.7793 0.9700 0.8278	0.9290 0.6736 0.9461 0.7934 0.9702 0.8393	0.9820 0.9219 0.9871 0.9655 0.9984 0.9540	0.9300 0.6610 0.9470 0.7933 0.9713 0.8360
LMCI/EMCI	EfficientNetB2 InceptionV3 RegNetx006 Basic ViT EfficientNetB2 + FPN (Our Model) ViT + EfficientNetB2 + FPN (Our Model)	0.9839 0.9481 0.9871 0.9695 0.9846 **0.9877**	0.9744 0.8692 0.9883 0.9294 0.9771 0.9861	0.9753 0.8516 0.9845 0.9174 0.9756 0.9820	0.9741 0.8594 0.9871 0.9237 0.9771 0.9840	0.9912 0.9771 0.9713 0.9723 0.9893 0.9932	0.9746 0.8597 0.9866 0.9235 0.9763 0.9843

**Table 7:** Classification results obtained when the models are run 1 vs. 1 (5 slices with SSIM).

1 vs. 1	Models	Accuracy	Precision	Recall	F1 Score	AUC	MCC
CN/AD	EfficientNetB2 InceptionV3 RegNetx006 Basic ViT EfficientNetB2 + FPN (Our Model) ViT + EfficientNetB2 + FPN (Our Model)	0.9756 0.9012 0.9858 0.9128 **0.9881** 0.9859	0.9473 0.7627 0.9673 0.7922 0.9718 0.9645	0.9423 0.8194 0.9612 0.7582 0.9688 0.9640	0.9446 0.7927 0.9670 0.7757 0.9720 0.9646	0.9838 0.9719 0.9882 0.9591 0.9982 0.9861	0.9491 0.8375 0.9702 0.8236 0.9734 0.9668
CN/MCI	EfficientNetB2 InceptionV3 RegNetx006 Basic ViT EfficientNetB2 + FPN (Our Model) ViT + EfficientNetB2 + FPN (Our Model)	0.9649 0.9283 **0.9891** 0.9172 0.9872 0.9788	0.9448 0.8289 0.9718 0.7983 0.9696 0.9469	0.9429 0.8194 0.9799 0.7681 0.9664 0.9469	0.9440 0.8267 0.9731 0.7824 0.9685 0.9469	0.9819 0.9519 0.9995 0.9675 0.9982 0.9711	0.9474 0.8299 0.9754 0.7903 0.9698 0.9469
CN/LMCI	EfficientNetB2 InceptionV3 RegNetx006 Basic ViT EfficientNetB2 + FPN (Our Model) ViT + EfficientNetB2 + FPN (Our Model)	0.9783 0.9192 0.9896 0.9189 0.9881 **0.9898**	0.9492 0.8122 0.9746 0.7997 0.9715 0.9797	0.9413 0.7895 0.9735 0.7817 0.9691 0.9794	0.9445 0.8019 0.9742 0.7906 0.9745 0.9795	0.9962 0.9691 0.9987 0.9781 0.9818 0.9822	0.9507 0.8008 0.9741 0.7923 0.9717 0.9795
CN/EMCI	EfficientNetB2 InceptionV3 RegNetx006 Basic ViT EfficientNetB2 + FPN (Our Model) ViT + EfficientNetB2 + FPN (Our Model)	0.9769 0.8983 0.9883 0.9247 0.9850 **0.9892**	0.9428 0.7712 0.9718 0.8283 0.9650 0.9728	0.9438 0.7912 0.9683 0.8012 0.9600 0.9382	0.9427 0.7812 0.9742 0.8140 0.9628 0.9548	0.9849 0.9591 0.9819 0.9699 0.9875 0.9884	0.9431 0.7806 0.9714 0.8144 0.9626 0.9529
AD/MCI	EfficientNetB2 InceptionV3 RegNetx006 Basic ViT EfficientNetB2 + FPN (Our Model) ViT + EfficientNetB2 + FPN (Our Model)	0.9819 0.9383 0.9881 0.9239 **0.9900** 0.9890	0.9592 0.8619 0.9891 0.8634 0.9864 0.9508	0.9521 0.8371 0.9721 0.7898 0.9737 0.9491	0.9540 0.8492 0.9795 0.8276 0.9798 0.9502	0.9920 0.9781 0.9961 0.9695 0.9986 0.9818	0.9567 0.8494 0.9820 0.8269 0.9800 0.9500
AD/LMCI	EfficientNetB2 InceptionV3 RegNetx006 Basic ViT EfficientNetB2 + FPN (Our Model) ViT + EfficientNetB2 + FPN (Our Model)	0.9449 0.9693 0.9942 0.9757 **0.9945** 0.9697	0.8544 0.9382 0.9809 0.9369 0.9868 0.9268	0.8529 0.9193 0.9891 0.9371 0.9856 0.9215	0.8515 0.9283 0.9799 0.9360 0.9850 0.9226	0.9733 0.9955 0.9973 0.9947 0.9988 0.9503	0.8529 0.9274 0.9850 0.9367 0.9861 0.9236
AD/EMCI	EfficientNetB2 InceptionV3 RegNetx006 Basic ViT EfficientNetB2 + FPN (Our Model) ViT + EfficientNetB2 + FPN (Our Model)	0.9895 0.9482 0.9822 0.9683 **0.9919** 0.9475	0.9742 0.8910 0.9810 0.9099 0.9813 0.8741	0.9739 0.8618 0.9701 0.9074 0.9780 0.8614	0.9740 0.8783 0.9748 0.9072 0.9791 0.8687	0.9917 0.9891 0.9946 0.9889 0.9988 0.9261	0.9739 0.8763 0.9753 0.9082 0.9791 0.8671
MCI/LMCI	EfficientNetB2 InceptionV3 RegNetx006 Basic ViT EfficientNetB2 + FPN (Our Model) ViT + EfficientNetB2 + FPN (Our Model)	0.9518 0.8973 0.9881 0.9181 **0.9891** 0.9887	0.9129 0.7672 0.9784 0.7896 0.9737 0.9621	0.9192 0.7221 0.9794 0.7724 0.9717 0.9612	0.9172 0.7435 0.9782 0.7797 0.9701 0.9626	0.9839 0.9719 0.9889 0.9691 0.9985 0.9808	0.9164 0.7442 0.9786 0.7806 0.9718 0.9619
MCI/EMCI	EfficientNetB2 InceptionV3 RegNetx006 Basic ViT EfficientNetB2 + FPN (Our Model) ViT + EfficientNetB2 + FPN (Our Model)	0.9749 0.8771 0.9795 0.9164 **0.9888** 0.9862	0.9317 0.7219 0.9484 0.8091 0.9739 0.8496	0.9311 0.6329 0.9463 0.7793 0.9700 0.8278	0.9290 0.6736 0.9461 0.7934 0.9702 0.8393	0.9820 0.9219 0.9871 0.9655 0.9984 0.9540	0.9300 0.6610 0.9470 0.7933 0.9713 0.8360
LMCI/EMCI	EfficientNetB2 InceptionV3 RegNetx006 Basic ViT EfficientNetB2 + FPN (Our Model) ViT + EfficientNetB2 + FPN (Our Model)	0.9839 0.9481 0.9871 0.9695 0.9846 **0.9877**	0.9744 0.8692 0.9883 0.9294 0.9771 0.9861	0.9753 0.8516 0.9845 0.9174 0.9756 0.9820	0.9741 0.8594 0.9871 0.9237 0.9771 0.9840	0.9912 0.9771 0.9713 0.9723 0.9893 0.9932	0.9746 0.8597 0.9866 0.9235 0.9763 0.9843

The z-scores for the architectures used in predicting Alzheimer’s disease are presented in Tables 12 and 13.

**Table 8:** The potential results of the two classifiers.

z score	Classifier A Failed	Classifier A Succeeded
Classifier B Failed	N_ff_	N_sf_
Classifier B Succeeded	N_fs_	N_ss_

**Table 9:** z scores and confidence levels.

z score	One-tailed prediction	Two-tailed prediction
1.345	95%	90%
1.960	97.5%	95%
2.326	99%	98%
2.576	99.5%	99%

**Table 10:** z-scores of architectures for manually selected slices in Alzheimer’s prediction.

1 vs. 1	CN vs. AD					
	EfficientNetB2	InceptionV3	RegNetx006	Basic ViT	EfficientNetB2 + FPN	ViT + EfficientNetB2 + FPN
EfficientNetB2		**←**0.891	**↑1.62E-36**	**↑**0.1372	**↑0.004**	**↑1.92E-16**
InceptionV3			**↑1.48E-36**	**↑**0.176	**↑0.007**	**↑1.72E-15**
RegNetx006				**←2**,**64E-32**	**←9**,**61E-25**	**←7.93E-08**
Basic ViT					**↑**0.183	**↑3.87E-12**
EfficientNetB2 + FPN						**↑3.05E-08**
ViT + EfficientNetB2 + FPN						
	CN vs. MCI					
EfficientNetB2		**←1.04E-14**	**↑0.0003**	**←2.27E-15**	**↑0.0002**	**←**0.948
InceptionV3			**↑1.03E-25**	**↑**0.579	**↑1.22E-26**	**↑2.17E-13**
RegNetx006				**←3.25E-28**	**←**1.000	**←0.0003**
Basic ViT					**↑2.22E-29**	**↑3.25E-15**
EfficientNetB2 + FPN						**←0.0003**
ViT + EfficientNetB2 + FPN						
	CN vs. LMCI					
EfficientNetB2		**←8.11E-07**	**↑7.93E-08**	**←1.30E-17**	**↑2.88E-13**	**↑3.80E-08**
InceptionV3			**↑2.77E-23**	**←7.29E-05**	**↑5.59E-30**	**↑2.98E-22**
RegNetx006				**←2.17E-38**	**↑0.022**	**←**1.000
Basic ViT					**↑4.26E-47**	**↑1.64E-40**
EfficientNetB2 + FPN						**←0.031**
ViT + EfficientNetB2 + FPN						
	CN vs. EMCI					
EfficientNetB2		**←0.0042**	**↑0.0106**	**←1.29E-12**	**↑0.016**	**←**0.0784
InceptionV3			**↑1.88E-08**	**←8.54E-06**	**↑6.64E-08**	**↑**0.2613
RegNetx006				**←9.05E-21**	**←**0.8474	**←1.85E-05**
Basic ViT					**↑7.42E-21**	**↑4.50E-08**
EfficientNetB2 + FPN						**←4.11E-05**
ViT + EfficientNetB2 + FPN						
	AD vs. MCI					
EfficientNetB2		**←0.0001**	**↑0.014**	**←3.02E-14**	**←0.0153**	**↑**0.1408
InceptionV3			**↑**0.1573	**←3.40E-25**	**↑**0.1573	**↑2.48E-07**
RegNetx006				**←3.58E-21**	**↑**1.0000	**←0.0002**
Basic ViT					**↑9.66E-21**	**↑4.20E-10**
EfficientNetB2 + FPN						**←1.72E-04**
ViT + EfficientNetB2 + FPN						
	AD vs. LMCI					
EfficientNetB2		**←0.0018**	**←**1.000	**←0.0184**	**↑**0.2568	**←0.0018**
InceptionV3			**↑0.0027**	**↑**0.3692	**↑1.24E-04**	**←**1.000
RegNetx006				**←0.0253**	**↑**0.2568	**←0.0027**
Basic ViT					**↑0.0016**	**←**0.3841
EfficientNetB2 + FPN						**←1.24E-04**
ViT + EfficientNetB2 + FPN						
	AD vs. EMCI					
EfficientNetB2		**←0.0002**	**↑**1.000	**←5.93E-06**	**←**1.000	**←6.44E-04**
InceptionV3			**↑2.57E-04**	**←**0.2850	**↑1.62E-04**	**↑**0.7773
RegNetx006				**←5.93E-06**	**←**1.000	**←6.44E-04**
Basic ViT					**↑5.93E-06**	**↑**0.1814
EfficientNetB2 + FPN						**←0.0006**
ViT + EfficientNetB2 + FPN						
	MCI vs. LMCI					
EfficientNetB2		**←3.06E-04**	**↑2.59E-04**	**←2.68E-08**	**↑0.024**	**←**0.9136
InceptionV3			**↑4.18E-12**	**←0.0287**	**←7.76E-09**	**↑4.07E-04**
RegNetx006				**←1.70E-17**	**←**0.1404	**←3.36E-04**
Basic ViT					**↑4.74E-15**	**↑2.09E-08**
EfficientNetB2 + FPN						**←0.0201**
ViT + EfficientNetB2 + FPN						
	MCI vs. EMCI					
EfficientNetB2		**←2.30E-12**	**↑0.0330**	**←0.0330**	**↑1.23E-06**	**←**0.6662
InceptionV3			**↑6.94E-17**	**↑1.34E-17**	**↑2.01E-26**	**↑4.71E-12**
RegNetx006				**←**1.000	**↑0.0047**	**←0.0133**
Basic ViT					**↑0.0039**	**↑0.0123**
EfficientNetB2 + FPN						**←2.15E-07**
ViT + EfficientNetB2 + FPN						
	LMCI vs. EMCI					
EfficientNetB2		**←0.0011**	**↑**0.7389	**←0.0060**	**↑**0.7630	**←**0.3173
InceptionV3			**↑0.0011**	**↑**0.7389	**↑0.0011**	**↑0.0339**
RegNetx006				**←0.0018**	**↑**1.000	**↑**0.1655
Basic ViT					**↑0.0027**	**↑**0.0588
EfficientNetB2 + FPN						**←**0.1967
ViT + EfficientNetB2 + FPN						

**Table 11:** z-scores of architectures for with SSIM selected slices in Alzheimer’s prediction.

1 vs. 1	CN vs. AD					
	EfficientNetB2	InceptionV3	RegNetx006	Basic ViT	EfficientNetB2 + FPN	ViT + EfficientNetB2 + FPN
EfficientNetB2		**←3.01E-24**	**↑**0.2918	**←3.42E-21**	**↑**0.3657	**↑**0.3074
InceptionV3			**↑1.60E-28**	**↑**0.2971	**↑1.54E-27**	**↑2.55E-28**
RegNetx006				**←2**,**99E-24**	**↑**0.9165	**↑**1.000
Basic ViT					**↑4.91E-26**	**↑5.27E-25**
EfficientNetB2 + FPN						**←**0.9146
ViT + EfficientNetB2 + FPN						
	CN vs. MCI					
EfficientNetB2		**←3.67E-13**	**↑1.42E-07**	**←5.03E-23**	**↑1.42E-07**	**↑**0.1093
InceptionV3			**↑4.76E-32**	**←**0.0656	**↑2.90E-33**	**↑5.54E-19**
RegNetx006				**←3.06E-40**	**←**1.000	**←1.02E-04**
Basic ViT					**↑3.06E-40**	**↑4.79E-26**
EfficientNetB2 + FPN						**↑7.48E-05**
ViT + EfficientNetB2 + FPN						
	CN vs. LMCI					
EfficientNetB2		**←5.37E-16**	**↑0.033**	**←2.56E-16**	**↑0.026**	**↑0.026**
InceptionV3			**↑2.82E-22**	**↑**0.9123	**↑1.14E-22**	**↑1.67E-23**
RegNetx006				**←6.92E-22**	**↑**0.9042	**↑**0.9042
Basic ViT					**↑2.81E-22**	**↑1.10E-22**
EfficientNetB2 + FPN						**↑**1.000
ViT + EfficientNetB2 + FPN						
	CN vs. EMCI					
EfficientNetB2		**←1.11E-29**	**↑0.0014**	**←4.07E-13**	**↑6.82E-04**	**↑8.12E-04**
InceptionV3			**↑1.76E-39**	**↑6.61E-05**	**↑3.59E-41**	**↑5.84E-41**
RegNetx006				**←1.01E-24**	**←**1.000	**↑**0.898
Basic ViT					**↑4.18E-23**	**↑6.62E-23**
EfficientNetB2 + FPN						**↑**0.884
ViT + EfficientNetB2 + FPN						
	AD vs. MCI					
EfficientNetB2		**←2.02E-24**	**↑**1.000	**←1.10E-13**	**↑**0.0679	**↑**1.000
InceptionV3			**↑5.47E-25**	**←6.02E-04**	**↑1.09E-28**	**↑1.06E-24**
RegNetx006				**←2.81E-13**	**↑**0.0588	**↑**1.000
Basic ViT					**↑5.04E-19**	**↑1.78E-13**
EfficientNetB2 + FPN						**←**0.0679
ViT + EfficientNetB2 + FPN						
	AD vs. LMCI					
EfficientNetB2		**↑**0.1172	**↑4.18E-04**	**↑0.0133**	**↑2.56E-04**	**↑**0.1282
InceptionV3			**↑0.0253**	**↑**0.2971	**↑0.0184**	**←**1.000
RegNetx006				**←**0.1967	**↑**1.000	**←0.0184**
Basic ViT					**↑**0.1655	**←**0.2971
EfficientNetB2 + FPN						**←0.0253**
ViT + EfficientNetB2 + FPN						
	AD vs. EMCI					
EfficientNetB2		**←6.33E-05**	**←0.0143**	**←0.0028**	**↑**0.763	**←1.86E-06**
InceptionV3			**↑4.32E-08**	**↑**0.1489	**↑2.38E-05**	**←**0.5287
RegNetx006				**←7.74E-06**	**↑0.0253**	**←3.30E-09**
Basic ViT					**↑0.0027**	**←0.0431**
EfficientNetB2 + FPN						**←1.14E-06**
ViT + EfficientNetB2 + FPN						
	MCI vs. LMCI					
EfficientNetB2		**←2.68E-07**	**↑2.75E-04**	**←3.09E-05**	**↑3.36E-04**	**↑6.64E-08**
InceptionV3			**↑6.36E-16**	**↑**0.4817	**↑2.94E-15**	**↑**0.4982
RegNetx006				**←2.81E-13**	**↑**1.000	**↑5.69E-18**
Basic ViT					**↑4.05E-14**	**↑**0.1552
EfficientNetB2 + FPN						**←2.87E-17**
ViT + EfficientNetB2 + FPN						
	MCI vs. EMCI					
EfficientNetB2		**←**0.1037	**↑0.0047**	**←1.47E-09**	**↑0.0047**	**↑**0.9055
InceptionV3			**↑7.42E-06**	**↑1.85E-06**	**↑1.42E-05**	**↑**0.1311
RegNetx006				**←2.30E-18**	**↑**1.000	**↑0.0033**
Basic ViT					**↑2.11E-16**	**↑6.76E-10**
EfficientNetB2 + FPN						**←0.0033**
ViT + EfficientNetB2 + FPN						
	LMCI vs. EMCI					
EfficientNetB2		**←3.47E-04**	**↑**0.7389	**←0.0290**	**↑**0.7389	**↑0.0011**
InceptionV3			**↑0.0027**	**↑**0.1779	**↑0.0027**	**↑**1.0000
RegNetx006				**←**0.0593	**←**1.0000	**↑0.0027**
Basic ViT					**↑**0.0593	**↑**0.2230
EfficientNetB2 + FPN						**↑0.0027**
ViT + EfficientNetB2 + FPN						

Tables 10 and 11 display a performance comparison of various classifiers on the dataset, using arrowheads (←, ↑) to highlight which classifier achieved superior results regarding true predictions (both true positives and negatives). Accompanying the arrowheads are z-scores, indicating the statistical significance of these differences. Bold font is used in the tables to emphasize statistically significant outcomes. If significance is found, the confidence levels for one-tailed and two-tailed predictions are listed below.

Table 10 shows the classification results of manually selected slices for 1 vs. 1 classifications of 6 deep learning architectures. ViT + EfficientNetB2 + FPN and EfficientNetB2 + FPN are the best performing models for Alzheimer’s disease classification. The z-score values in the tables reveal that this model shows very high statistical significance for each classification task. The success of this model can be explained by combining the powerful feature extraction capacity of EfficientNetB2 with the attention mechanism of Vision Transformer (ViT). Moreover, its efficient processing of multilayer features with FPN provides a great advantage in complex classification problems such as Alzheimer’s disease.

Table 11 shows the classification results of slices selected with SSIM for 1 vs. 1 classifications of 6 deep learning architectures. The ViT + EfficientNetB2 + FPN model is the best performing model for Alzheimer’s disease classification. ViT + EfficientNetB2 + FPN model can be said to be the best model for Alzheimer’s classification. The ViT + EfficientNetB2 + FPN model shows top performance on challenging classification tasks, while the EfficientNetB2 + FPN model is a highly competitive alternative. In terms of statistical significance, both models are far above the other models and can be considered as the two most reliable models for accurately classifying the different stages of Alzheimer’s disease.

In addition, training time, model size, and inference speed information for the models run are given in Tables 12, 13, 14 and 15. Tables 14 and 15 include the training times in the 1 vs. 1 classification.

**Table 12:** z-scores of architectures for manually selected slices in Alzheimer’s prediction.

Models	Parameter numbers	Model size	Inference speed		Estimated epoch duration	Total training time
EfficientNetB2 InceptionV3 RegNetx006 Basic ViT EfficientNetB2 + FPN (Our Model) ViT + EfficientNetB2 + FPN (Our Model)	9.2 M 23.9 M 6.2 M 86.0 M 9.5 M 95 M	8 MB 92 MB 120 MB 200 MB 15 MB 250 MB		50ms 100ms 40ms 200ms 80ms 250ms	8 min 12 min 7 min 18 min 10 min 20 min	1.3 h 2.0 h 1.2 h 3.0 h 1.7 h 3.3 h

**Table 13:** z-scores of architectures for with SSIM selected slices in Alzheimer’s prediction.

Models	Parameter numbers	Model size	Inference speed		Estimated epoch duration	Total training time
EfficientNetB2 InceptionV3 RegNetx006 Basic ViT EfficientNetB2 + FPN (Our Model) ViT + EfficientNetB2 + FPN (Our Model)	9.2 M 23.9 M 6.2 M 86.0 M 9.5 M 95 M	8 MB 92 MB 120 MB 200 MB 15 MB 250 MB		50ms 100ms 40ms 200ms 80ms 250ms	7.2 min 10.8 min 6.3 min 16.2 min 9.0 min 18 min	1.2 h 1.8 h 1.1 h 2.7 h 1.5 h 3.0 h

**Table 14:** Training times with 1 vs. 1 (5 slices selected manually).

1 vs. 1	Models	Estimated epoch duration	Total training time
CN/AD	EfficientNetB2 InceptionV3 RegNetx006 Basic ViT EfficientNetB2+FPN (Our Model) ViT + EfficientNetB2 + FPN (Our Model)	39 seconds 52 seconds 46 seconds 65 seconds 78 seconds 98 seconds	6.5 minutes 8.7 minutes 7.6 minutes 10.8 minutes 13 minutes 16.3 minutes
CN/MCI	EfficientNetB2 InceptionV3 RegNetx006 Basic ViT EfficientNetB2+FPN (Our Model) ViT + EfficientNetB2 + FPN (Our Model)	49 seconds 65 seconds 58 seconds 82 seconds 98 seconds 123 seconds	8.2 minutes 10.9 minutes 9.6 minutes 13.7 minutes 16.4 minutes 20.5 minutes
CN/LMCI	EfficientNetB2 InceptionV3 RegNetx006 Basic ViT EfficientNetB2+FPN (Our Model) ViT + EfficientNetB2 + FPN (Our Model)	36 seconds 47 seconds 42 seconds 59 seconds 71 seconds 89 seconds	6 minutes 7.9 minutes 7.0 minutes 9.9 minutes 11.9 minutes 14.9 minutes
CN/EMCI	EfficientNetB2 InceptionV3 RegNetx006 Basic ViT EfficientNetB2+FPN (Our Model) ViT + EfficientNetB2 + FPN (Our Model)	38 seconds 50 seconds 44 seconds 63 seconds 76 seconds 95 seconds	6.3 minutes 8.4 minutes 7.4 minutes 10.5 minutes 12.6 minutes 15.8 minutes
AD/MCI	EfficientNetB2 InceptionV3 RegNetx006 Basic ViT EfficientNetB2+FPN (Our Model) ViT + EfficientNetB2 + FPN (Our Model)	20 seconds 26 seconds 23 seconds 33 seconds 40 seconds 50 seconds	3.3 minutes 4.4 minutes 3.85 minutes 5.5 minutes 6.6 minutes 8.25 minutes
AD/LMCI	EfficientNetB2 InceptionV3 RegNetx006 Basic ViT EfficientNetB2+FPN (Our Model) ViT + EfficientNetB2 + FPN (Our Model)	6 seconds 8 seconds 7 seconds 11 seconds 13 seconds 16 seconds	1.05 minutes 1.4 minutes 1.2 minutes 1.75 minutes 2.1 minutes 2.6 minutes
AD/EMCI	EfficientNetB2 InceptionV3 RegNetx006 Basic ViT EfficientNetB2+FPN (Our Model) ViT + EfficientNetB2 + FPN (Our Model)	8 seconds 11 seconds 10 seconds 14 seconds 17 seconds 21 seconds	1.4 minutes 1.87 minutes 1.63 minutes 2.33 minutes 2.8 minutes 3.5 minutes
MCI/LMCI	EfficientNetB2 InceptionV3 RegNetx006 Basic ViT EfficientNetB2+FPN (Our Model) ViT + EfficientNetB2 + FPN (Our Model)	17 seconds 22 seconds 19 seconds 28 seconds 33 seconds 41 seconds	2.75 minutes 3.67 minutes 3.2 minutes 4.58 minutes 5.5 minutes 6.88 minutes
MCI/EMCI	EfficientNetB2 InceptionV3 RegNetx006 Basic ViT EfficientNetB2+FPN (Our Model) ViT + EfficientNetB2 + FPN (Our Model)	19 seconds 25 seconds 22 seconds 31 seconds 37 seconds 47 seconds	3.1 minutes 4.13 minutes 3.62 minutes 5.17 minutes 6.2 minutes 7.75 minutes
LMCI/EMCI	EfficientNetB2 InceptionV3 RegNetx006 Basic ViT EfficientNetB2+FPN (Our Model) ViT + EfficientNetB2 + FPN (Our Model)	5 seconds 7 seconds 6 seconds 8 seconds 10 seconds 13 seconds	0.85 minutes 1.13 minutes 1.0 minutes 1.42 minutes 1.7 minutes 2.1 minutes

**Table 15:** Training times with 1 vs. 1 (5 slices selected with SSIM).

1 vs. 1	Models	Estimated epoch duration	Total training time
CN/AD	EfficientNetB2 InceptionV3 RegNetx006 Basic ViT EfficientNetB2+FPN (Our Model) ViT + EfficientNetB2 + FPN (Our Model)	33 seconds 47 seconds 39 seconds 60 seconds 71 seconds 90 seconds	5.8 minutes 7.5 minutes 6.2 minutes 8.3 minutes 12 minutes 15.1 minutes
CN/MCI	EfficientNetB2 InceptionV3 RegNetx006 Basic ViT EfficientNetB2+FPN (Our Model) ViT + EfficientNetB2 + FPN (Our Model)	41 seconds 59 seconds 51 seconds 77 seconds 85 seconds 119 seconds	7.1 minutes 8.5 minutes 8.7 minutes 12.5 minutes 15.3 minutes 19.3 minutes
CN/LMCI	EfficientNetB2 InceptionV3 RegNetx006 Basic ViT EfficientNetB2+FPN (Our Model) ViT + EfficientNetB2 + FPN (Our Model)	31 seconds 39 seconds 38 seconds 49 seconds 69 seconds 75 seconds	4.8 minutes 6.4 minutes 6.3 minutes 8.4 minutes 10.4 minutes 13.6 minutes
CN/EMCI	EfficientNetB2 InceptionV3 RegNetx006 Basic ViT EfficientNetB2+FPN (Our Model) ViT + EfficientNetB2 + FPN (Our Model)	38 seconds 50 seconds 44 seconds 63 seconds 76 seconds 95 seconds	5.3 minutes 7.2 minutes 6.1 minutes 9.6 minutes 11.4 minutes 14.3 minutes
AD/MCI	EfficientNetB2 InceptionV3 RegNetx006 Basic ViT EfficientNetB2+FPN (Our Model) ViT + EfficientNetB2 + FPN (Our Model)	18 seconds 22 seconds 16 seconds 25 seconds 30 seconds 45 seconds	2.1 minutes 3.4 minutes 2.6 minutes 4.3 minutes 5.6 minutes 7.5 minutes
AD/LMCI	EfficientNetB2 InceptionV3 RegNetx006 Basic ViT EfficientNetB2+FPN (Our Model) ViT + EfficientNetB2 + FPN (Our Model)	5 seconds 7 seconds 7 seconds 8 seconds 11 seconds 12 seconds	1.01 minutes 1.2 minutes 1.1 minutes 1.43 minutes 1.8 minutes 1.6 minutes
AD/EMCI	EfficientNetB2 InceptionV3 RegNetx006 Basic ViT EfficientNetB2+FPN (Our Model) ViT + EfficientNetB2 + FPN (Our Model)	7 seconds 9 seconds 9 seconds 11 seconds 16 seconds 18 seconds	1.2 minutes 1.56 minutes 1.43 minutes 1.29 minutes 1.5 minutes 2.6 minutes
MCI/LMCI	EfficientNetB2 InceptionV3 RegNetx006 Basic ViT EfficientNetB2+FPN (Our Model) ViT + EfficientNetB2 + FPN (Our Model)	15 seconds 17 seconds 13 seconds 18 seconds 27 seconds 38 seconds	2.43 minutes 2.52 minutes 2.8 minutes 3.21 minutes 4.2 minutes 5.91 minutes
MCI/EMCI	EfficientNetB2 InceptionV3 RegNetx006 Basic ViT EfficientNetB2+FPN (Our Model) ViT + EfficientNetB2 + FPN (Our Model)	15 seconds 18 seconds 20 seconds 28 seconds 29 seconds 38 seconds	3.1 minutes 4.13 minutes 3.62 minutes 5.17 minutes 6.2 minutes 7.75 minutes
LMCI/EMCI	EfficientNetB2 InceptionV3 RegNetx006 Basic ViT EfficientNetB2+FPN (Our Model) ViT + EfficientNetB2 + FPN (Our Model)	4 seconds 6 seconds 6 seconds 7 seconds 9 seconds 11 seconds	0.75 minutes 1.10 minutes 1.0 minutes 1.32 minutes 1.67 minutes 2.0 minutes

When comparing Tables 12 and 13, it is observed that training with manually selected slices (Table 12) results in longer epoch durations and total training times for all models compared to training with SSIM-selected slices (Table 13). Notably, models trained with manual selection exhibit higher epoch and total training durations than those trained with SSIM-based selection, with the most significant differences observed in the Basic ViT and ViT + EfficientNetB2 + FPN models. For instance, the total training time for the Basic ViT model is 3.0 h with manually selected slices, whereas it decreases to 2.7 h with SSIM-selected slices. Similarly, the ViT + EfficientNetB2 + FPN model’s training time decreases from 3.3 to 3.0 h. This suggests that the SSIM-based slice selection method optimizes data representation, leading to a more efficient training process.

Tables 14 and 15 present training times for pairwise (1 vs. 1) classification tasks using five slices, selected either manually (Table 14) or via the SSIM-based method (Table 15). Both tables show that training time increases with model complexity, especially for models incorporating FPN and ViT components. However, training times in Table 15, where SSIM-selected slices were used, are generally shorter or comparable to those in Table 14. This suggests the SSIM method may lead to more informative and homogeneous slice selection, enabling more efficient learning. For example, in the CN vs. AD classification, the ViT + EfficientNetB2 + FPN model takes 16.3 min to train with manually selected slices, whereas it only takes 15.1 min with SSIM-selected slices. Similar reductions in training time can be observed in other class pairs, such as AD vs. MCI and MCI vs. LMCI. These findings indicate that SSIM-based slice selection may also improve model performance and contribute to more efficient training processes.

**Fig. 8:**
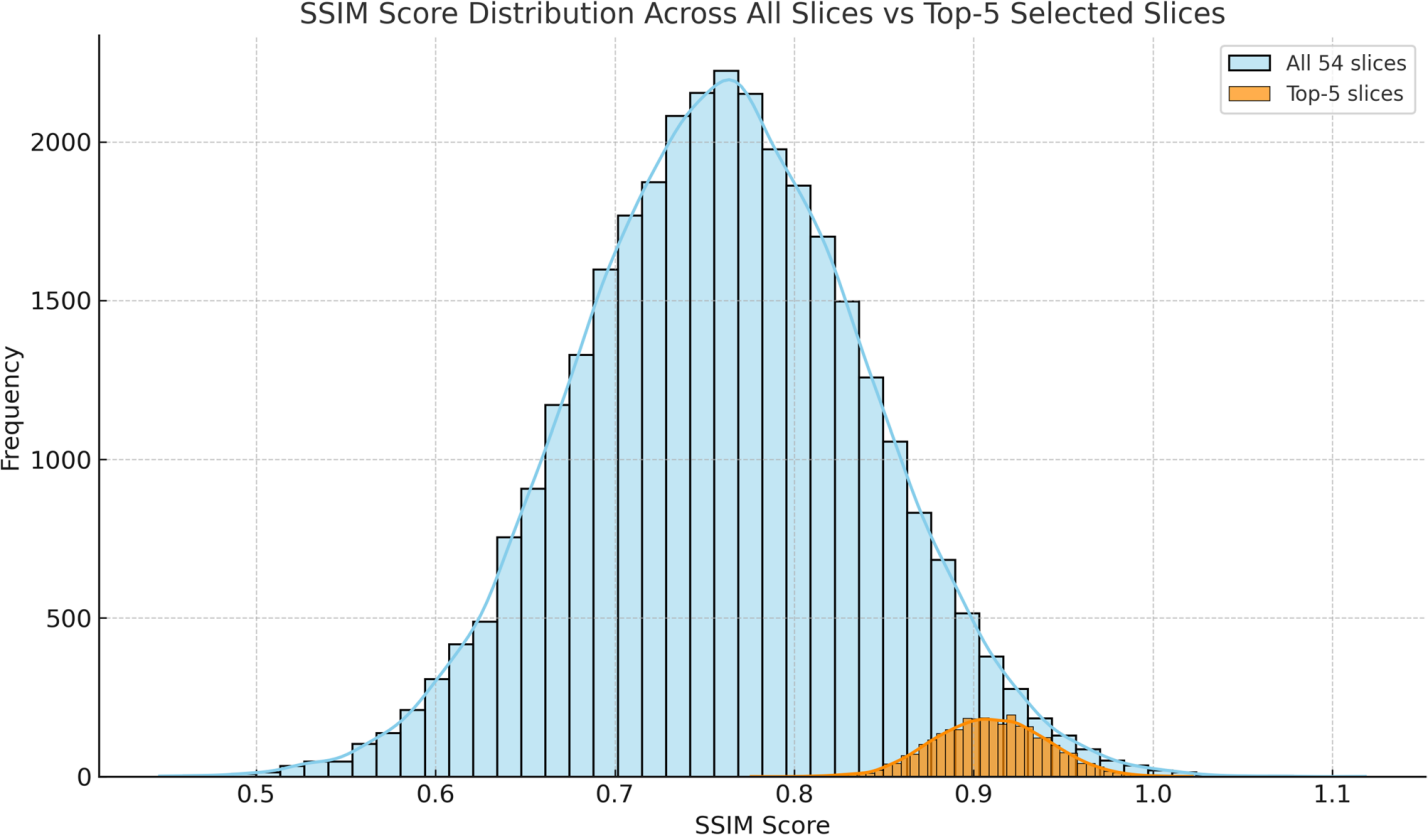
Distribution of SSIM Scores Across All Slices and Top-5 Selected Slices.

To justify the use of top-5 slice selection without applying a fixed threshold, the distribution of SSIM scores across all slices was analyzed. The blue curve and bars represent the SSIM score distribution for all slices (54 × number of patients), with a mean of approximately 0.76. The orange curve and bars show the SSIM scores of only the top-5 slices, with a higher mean of roughly 0.91. As shown in Fig. 8, while SSIM scores exhibit a wide distribution across all slices, the top-5 slices demonstrate the highest similarity. Therefore, selection based on ranking rather than a fixed threshold is more meaningful.

The primary goal here is to show the importance of slice selection in AI-based Alzheimer’s diagnosis. When Alzheimer’s studies are analyzed, it is seen that slice selection has not been emphasized much. Generally, either all slices were used, or these processes were carried out by expert selection, so it is thought that the study has an important place in slice selection. One of the key points of the study is that instead of analyzing all slices, selecting only the necessary and meaningful slices reduced the data processing time and made the analysis more efficient. This also reduced the computational burden and the need for storage.

Apart from the slice selection described, the two different models proposed are believed to be an important contribution to the literature. Compared to other studies, Duan et al.^5^, obtained an accuracy of 89.58% for AD vs. CN, while we reached an accuracy of 98.81% in the EfficientNetB2 + FPN model with SSIM. Gamal et al.^10^, obtained 78.60% for AD vs. MCI with CNN + ViT, while in another recent study Şener et al.^11^, we obtained 97.95% for AD vs. MCI with the EfficientNetB0 model. In our current study, we outperformed the two studies with 99.00% accuracy rate using EfficientNetB2 + FPN, and 98.90% accuracy rate using ViT + EfficientNetB2 + FPN for AD vs. MCI. For CN vs. MCI, Gamal et al.^10^, achieved an accuracy of 78.86%, for CN vs. MCI an accuracy of 98.42% with the DenseNet121 model, while a more recent model such as RegNetx006 achieved an accuracy of 98.91%. In AD vs. EMCI classification, 99.19% accuracy was achieved with EfficientNetB2 + FPN, and at this point, detecting this at an early stage before the patient progressed to AD was among the goals we set.

The results were also significant according to McNemar’s statistical test. It can be said that both ViT + EfficientNetB2 + FPN and EfficientNetB2 + FPN models are the best models for Alzheimer’s classification. In terms of statistical significance, both models are above the other models and can be considered as the two most reliable models for accurately classifying different stages of Alzheimer’s disease. Compared to the literature, we can say that we obtained quite promising results in 1 vs. 1 classification results. It is believed that the reason for yielding such satisfactory results come from both the use of improved models and the novel slice selection approach.

For the generalizability of the proposed model, the proposed model is tested on the OASIS dataset. MRI images in AD and CN classes from the OASIS dataset were obtained. There are 150 objects in the OASIS dataset, of which 72 belong to CN and 78 to AD class. There are 128 T1-weighted slices for each object. Manually and in the same way, 5 slices were selected with SSIM. Then, the models we proposed were run and the results were obtained. The results are presented in Table 16, where we observe that the proposed model provides a slight performance improvement over SSIM on the manually selected slices and only slightly outperforms SSIM on the SSIM selected slices. This finding shows that our model can provide very robust and stable results.

**Table 16:** Classification results obtained when the models are run with OASIS dataset.

1 vs. 1	Models	Accuracy	Precision	Recall	F1 Score	AUC	MCC
(5 slices selected manually)							
CN/AD	EfficientNetB2 + FPN (Our Model) ViT + EfficientNetB2 + FPN (Our Model)	0.9165 **0.9571**	0.9390 0.8921	0.9372 0.8829	0.9345 0.8835	0.9654 0.9693	0.9341 0.8828
(5 slices selected SSIM)							
CN/AD	EfficientNetB2 + FPN (Our Model) ViT + EfficientNetB2 + FPN (Our Model)	0.9783 **0.9824**	0.9671 0.9592	0.9598 0.9525	0.9755 0.9630	0.9890 0.9864	0.9735 0.9654

## Conclusion

Alzheimer’s disease has recently become an increasing health problem. Diagnosing the disease at an early stage can help preserve cognitive function, enabling patients to carry out activities of daily living independently for longer. From these points of view, early diagnosis becomes a crucial situation. This study focuses on deep learning models, ViT and newly proposed models based on early detection of Alzheimer’s disease. The models used were selected based on current models. The main justification for choosing these models is that they provide a significant advantage, especially in rapidly changing fields (medicine, technology, etc.). In addition, with the new models we have proposed, an innovation has been brought over the current models. In addition, the importance of slice selection is also emphasized. To do that SSIM was used to select the best possible slices. And to make a comparison, the slices were first selected manually and then also selected with SSIM. In the manually selected slices, 5 slices were selected from the mid-region of the MRI sequence. In SSIM-based automatic selection, the first step was to determine a reference image for each class. Since our study obtained results on a dataset containing T1-weighted axial MRI images, it has the limitation of being on these images. When determining the reference image, the MRI slices for each patient were analyzed and the image with the largest number of edge segments among these slices was found and selected. When finding the reference image for a single class, a reference image was determined for each patient and finally a generic reference image with the largest edge was selected from the reference images.

The findings highlight the time-saving advantage of the SSIM-based slice selection method. Across all models and classification tasks, training durations—per epoch and total—were consistently shorter when slices were selected using SSIM rather than manual methods. For example, in the CN vs. AD classification using the ViT + EfficientNetB2 + FPN model, SSIM-based selection reduced training time by 1.2 min compared to manual selection. While the absolute reductions in time may appear modest, they become increasingly significant when scaled across multiple models, folds, and experiments. These results suggest that SSIM selection maintains informative content and supports a more time-efficient and scalable training process.

To evaluate the generalizability of the proposed model, additional experiments were conducted on the OASIS dataset, which includes 150 subjects (72 CN and 78 AD), each with 128 T1-weighted MRI slices. In alignment with the main study, five slices per subject were manually and automatically selected using the SSIM-based method. The proposed models were then applied to these selections, and the results are presented in Table 16. The results show that the proposed model achieves a slight performance improvement over SSIM when evaluated on manually selected slices and only slightly outperforms our result on slices selected by SSIM. These results emphasize the robustness and stability of the proposed approach on different datasets and strengthen its potential for reliable use in real-world clinical scenarios.

These efforts provide important contributions to the early detection of Alzheimer’s disease and are considered to provide a strong support for clinical diagnosis. In future studies, it is aimed to exceed these results with new model proposals by being aware of the importance of slice selection. The model will be validated on different datasets extensively with similar characteristics and some special methods are considered to be used for the unbalanced data set.

## Data Availability

The following information was supplied regarding data availability: The data is available from ADNI (https://adni.loni.usc.edu). The data is available at GitHub: https://github.com/AlzheimersDiseasebs/ADNI-3.
